# It is time to start taking tobacco seriously as a risk factor for psychosis: self‐medication cannot explain the association

**DOI:** 10.1111/acps.12923

**Published:** 2018-06-29

**Authors:** J. H. MacCabe

**Affiliations:** ^1^ Department of Psychosis Studies Institute of Psychiatry, Psychology and Neuroscience Kings College London London UK

The association between tobacco smoking and psychosis has long been recognised 1. Smoking has also found to be associated with subclinical psychotic symptoms in the general population 2, 3.

Until recently, it was generally assumed that the association was explained by reverse causation: that cigarette smoking was a consequence of the illness, whether through self‐medication to alleviate psychotic symptoms or the adverse effects of antipsychotics, or through a process of institutionalisation, whereby the smoking habit was culturally transmitted via hospitals and other mental healthcare settings. While cannabis has long been taken seriously as a potential causative agent for psychosis, the idea that tobacco might have a causative role has been largely ignored. But perhaps we should not be so ready to dismiss the possibility that this highly addictive psychoactive drug, often consumed during a critical phase of neurodevelopment, and with much greater regularity than most street drugs, could act as a risk factor for psychosis. True, the psychoactive effects of tobacco are mild and do not resemble psychosis, but we know that the effects of acute intoxication of a drug are a poor guide to its longer term associations with mental disorders; for instance, hallucinogens induce psychosis‐like states acutely but their effects on long‐term risk for psychosis are modest compared to those of other illicit drugs 4.

Like cannabis, tobacco is a challenging risk factor to study. The direction of causation is often unclear, especially in cross‐sectional or case–control studies. Furthermore, confounding is difficult to rule out. Many of the established social risk factors for psychotic disorders, such as social adversity and childhood trauma, are also associated with smoking. To further complicate matters, cannabis and tobacco smoking are highly correlated with one another, and also with other substance use 5, and while many cigarette smokers do not use cannabis, almost all cannabis users smoke tobacco, in cigarettes and/or as a component of cannabis joints 6.

The study of Mustonen et al. 7 provides important new evidence which can help us to disentangle these questions. Using data from the North Finland 1986 cohort, the authors studied associations between smoking habit at age 15–16 and risk of psychosis up to age 30.

Compared to non‐smokers, adolescents who smoked more than 10 cigarettes a day at age 15‐16 were around three times more likely to subsequently develop a psychotic disorder by age 30 (HR = 3.15; 95% CI: 1.94–5.13). While the time lag between the measurement of smoking and the onset of psychosis makes reverse causation unlikely, it remains possible that some individuals were experiencing psychotic symptoms at baseline, so self‐medication could have contributed to the association. One of the unique advantages of this study is that psychosis‐like experiences were assessed at baseline, allowing the authors to adjust for them; this adjustment resulted in only a small attenuation of the association between smoking and later psychotic disorder (HR = 2.87; 1.76–4.68), indicating that reverse causation was unlikely to be contributing substantially to the association.

If the association between smoking and psychosis was driven by self‐medication, we would expect the association between smoking and psychosis to be seen primarily in people who started smoking later, given the rarity of psychotic symptoms before age 15. In fact, the opposite was the case. Participants who first smoked before age 13 had more than twice the risk of psychosis than those who started smoking later, and moreover, this difference was not attenuated after adjustment for psychotic symptoms, cannabis use and other confounders. The stronger association in people who started smoking younger has been seen in other studies 8 is analogous to the similar effect observed with cannabis 9 and is consistent with a disrupting effect of smoking on neurodevelopmental processes during adolescence. It is equally consistent with earlier neurodevelopmental, genetic or psychosocial risk factors leading to an atypical developmental trajectory characterised by earlier smoking and, subsequently, an increased risk of psychosis in adulthood. It is not, however, consistent with reverse causation.

To assess the impact of confounding, the authors were able to adjust for parental psychosis, parental substance misuse, frequent alcohol misuse, other substance misuse and cannabis use. Of these, there was evidence of confounding by cannabis use, but in the fully adjusted model, the association between cigarette smoking and psychosis persisted (HR = 2.00; 1.13–3.54). However, due to the high collinearity between cannabis and tobacco smoking, more elaborate statistical techniques such as penalised regression may be needed to tease out the relative contributions of tobacco and cannabis to risk for psychosis. Furthermore, it is difficult to adequately adjust for all the psychosocial confounders, so residual confounding remains a possibility.

So what can we conclude about the association between tobacco smoking and risk for psychosis? This study provides arguably the clearest evidence to date that self‐medication and other reverse causation explanations cannot account for the association. That is not to say that self‐medication does not occur, but that it cannot explain away the association seen here. This is important, because self‐medication remains the dominant explanatory model among clinicians.

Whether tobacco has a direct causal impact on risk for psychosis, or whether the association is confounded by cannabis use or by other social or biological factors, remains an open question.

Whether or not it is causal, smoking can now be added to the long list of adverse outcomes that are statistically associated with cigarette smoking. As such, it has the potential to be an important health message. There has been a sharp decline in cannabis consumption in the UK in the past two decades (European Drug Report 2015 retrieved 1/6/18 from http://www.emcdda.europa.eu/publications/edr/trends-developments/2015/online/start), during a time when the statistical association between cannabis and psychosis started to be widely publicised in the UK media. It is likely that the risk of psychosis in early adulthood might resonate more strongly with young people than the risk of heart disease or cancer many decades later.
